# From Extrapolation to Precision Chemical Hazard Assessment: The Ecdysone Receptor Case Study

**DOI:** 10.3390/toxics10010006

**Published:** 2021-12-27

**Authors:** Raquel Ruivo, João Sousa, Teresa Neuparth, Olivier Geffard, Arnaud Chaumot, L. Filipe C. Castro, Davide Degli-Esposti, Miguel M. Santos

**Affiliations:** 1 CIIMAR/CIMAR—Interdisciplinary Centre of Marine and Environmental Research, University of Porto, 4450-208 Matosinhos, Portugal; jsousa@ciimar.up.pt (J.S.); tneuparth@ciimar.up.pt (T.N.); filipe.castro@ciimar.up.pt (L.F.C.C.); 2 Department of Biology, FCUP—Faculty of Sciences, University of Porto, 4169-007 Porto, Portugal; 3 ICBAS—Institute of Biomedical Sciences Abel Salazar, University of Porto, 4050-313 Porto, Portugal; 4 Ecotoxicology Team, INRAE—Institut National de Recherche pour l’Agriculture, l’Alimentation et l’Environnement, Unité de Recherche RiverLy, F-69625 Villeurbanne, France; olivier.geffard@inrae.fr (O.G.); arnaud.chaumot@inrae.fr (A.C.); davide.degli-esposti@inrae.fr (D.D.-E.)

**Keywords:** invertebrates, endocrine disruption, nuclear receptors, hazard assessment

## Abstract

Hazard assessment strategies are often supported by extrapolation of damage probabilities, regarding chemical action and species susceptibilities. Yet, growing evidence suggests that an adequate sampling of physiological responses across a representative taxonomic scope is of paramount importance. This is particularly relevant for Nuclear Receptors (NR), a family of transcription factors, often triggered by ligands and thus, commonly exploited by environmental chemicals. Within NRs, the ligand-induced Ecdysone Receptor (EcR) provides a remarkable example. Long regarded as arthropod specific, this receptor has been extensively targeted by pesticides, seemingly innocuous to non-target organisms. Yet, current evidence clearly suggests a wider presence of EcR orthologues across metazoan lineages, with unknown physiological consequences. Here, we address the state-of-the-art regarding the phylogenetic distribution and functional characterization of metazoan EcRs and provide a critical analysis of the potential disruption of such EcRs by environmental chemical exposure. Using EcR as a case study, hazard assessment strategies are also discussed in view of the development of a novel “precision hazard assessment paradigm.

## 1. Introduction

Environmental hazard and risk assessment aims to identify how a given chemical affects organisms, populations, communities and, ultimately, the ecosystem [1]. Chemical structural similarities, likelihood of toxic action and species susceptibilities are common approaches used to support the estimation of the hazard profile of a chemical, including effective concentrations and outcomes [2,3]. However, experimental data is often derived from a limited set of species, often with a poor phylogenetic distribution. An increasing number of examples in the literature indicates that, depending on the molecular target, even closely related species may display different responses towards the same chemical [4]. Therefore, including representativeness of genomic backgrounds and physiological responses within and across taxa is paramount to increase the confidence in hazard estimation, fostering extrapolations [5]. A phylogenetically relevant taxonomic sampling is, in fact, crucial for adequate comparative analysis, facilitating the prediction, through homology inference, of adverse outcomes in non-tested species with homologous modes of action (MoAs) [6]. In contrast to many legacy pollutants, the hazard assessment of Endocrine Disrupting Chemicals (EDCs) raises additional challenges. EDCs defy traditional risk assessments, given the (a) lack of proper hazard characterization of the numerous listed compounds, (b) the frequently observed non-monotonical response and low dose subchronic effects, (c) the emphasis on timing and duration of exposure and (d) the latent or even transgenerational effects produced [7]. Alternatively, EDCs may disrupt hormone action through different or a combination of molecular targets and MoAs; many of the harmful effects resulting from EDCs exposure have been shown to be mediated by interaction with Nuclear Receptors (NRs). NRs are a collection of diverse transcription factors that participate in the homeostatic regulation of hormonal systems. Their diversity is also mirrored by their distinct molecular mechanisms, forming hormone ligand-dependent or independent monomers, homodimers or heterodimers. Their modular architecture, including DNA binding and ligand binding domains (DBD and LBD), coupled by a flexible hinge region, allows the translation of a ligand signal into a transcriptional response [8]. Yet, given their ligand-binding abilities, NRs serve also as primary targets to EDCs [6]. Although NRs have been recognized in all metazoan groups, proper identification and characterization is highly skewed towards specific animal lineages, notably vertebrates, impairing an adequate inference of adverse outcomes in the multitude of invertebrate taxa [6,9,10,11]. In fact, and despite representing the vast majority of animal species, invertebrates are still the most neglected groups regarding NRs identification, characterization and subsequent assessment of EDC-mediated disruption [6,9,12]. The Ecdysone receptor (EcR) represents a prime example of our fragmented knowledge. EcR, along with its obligate heterodimeric partner, the Retinoid X Receptor (RXR), or its insect homolog, Ultraspiracle (USP), regulates the expression of target genes though binding to regulatory DNA sequences or response elements [13,14]. From an historical standpoint, EcR was long described as arthropod-specific, binding arthropod ecdysteroids and controlling moulting, development and reproduction [15,16]. This apparent taxonomic specificity further fostered the use of this hormone/receptor couple as target for the development of insect-specific pesticides, with an apparent low toxicity towards off-target species [17]. Yet, the growth of available genomic resources, made possible by the outburst of novel sequencing technologies, has brought to light a different scenario, with the identification of orthologous EcRs outside arthropods [18,19,20,21,22,23,24]. Despite the wealth of novel phylogenetic evidence, hazard assessment strategies are still lagging behind the genomic revolution.

Thus, in the present work, we address the phylogenetic distribution of EcR and contrast such distribution with known activation profiles of this receptor across taxa. Given the prominent role of EcR as an insecticide target, particular attention will be given to current knowledge on the modulation of metazoan EcR by such environmental compounds. Knowledge gaps will be highlighted as well as the implications of such a body of knowledge for the hazard assessment of EDCs and contaminants of emerging concern (CEC).

## 2. Phylogenetic Distribution and Function: State-of-the Art

**Figure 1:**
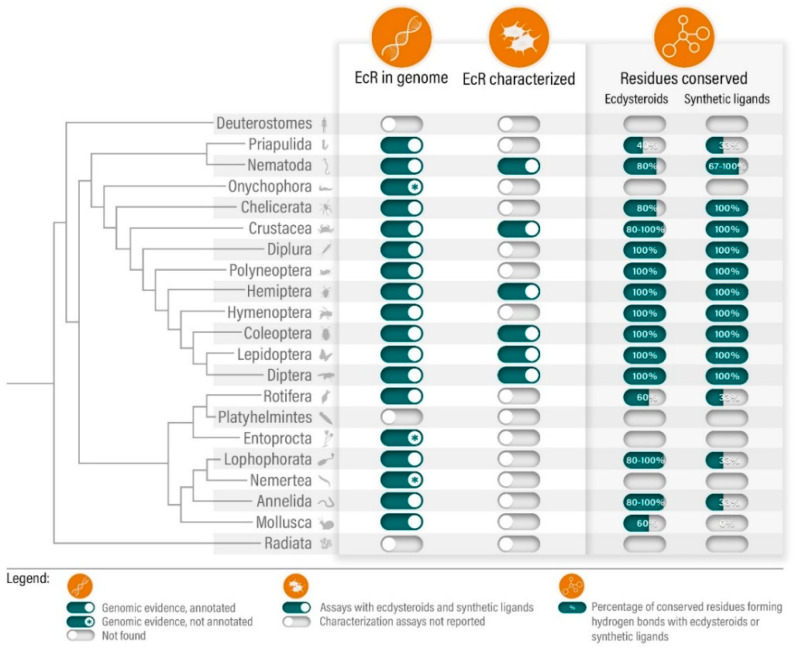
Summary of the state-of-the-art regarding the phylogenetic distribution and functional mapping of EcRs across metazoans.

While the first cloning and isolation of an EcR gene, from *Drosophila melanosgaster*, dates back to 1991, the occurrence of such receptors outside arthropods (Ecdysozoa) was only acknowledged in 2010: with the identification and cloning of EcR orthologues in nematodes (Ecdysozoa) [18,23,24] and, with the emergence of the genomes from the Lophotrochozoa *Lottia gigantea* (mollusc), *Helobdella robusta* (leech) and *Capitella teleta* (polychaete worm) [20]. The current growing collection of available genomic resources further expands this scenario. A simple search in the National Centre for Biotechnology Information (NCBI) resource database confirms that EcR gene annotations are widely present in both Ecdysozoan and Lophotrochozoan groups (Figure 1 and Table S1). Yet, while arthropod EcRs have been extensively associated with the expression of ecdysone-inducible genes, including other NRs (i.e., E75, HR3), modulating development, moulting and reproduction, the physiological role of non-arthropod orthologues in still unknown [14,15,25,26]. Additionally, and in spite of the wider phylogenetic distribution, functional characterization of EcRs with ecdysteroids (i.e., 20-hydroxyecdysone, Ponasterone A; [27]) is currently limited to insects and few crustaceans and nematodes: using ligand-binding assays or cell-based transactivation assays, assessing direct binding or transcriptional activity, respectively (Figure 1 and Table S2) [18,28,29,30,31,32,33,34,35,36,37,38]. The available data suggests that all tested EcRs respond to naturally occurring ecdysteroids, notably Ponasterone A, a potent EcR inducer and most commonly used steroid for EcR activity assessment. Concerning non-ecdysozoan EcRs, experimental information is absent, although comparative sequence and structural analysis (i.e., homology modelling and molecular docking analysis) could provide hints towards the functional status of non-ecdysozoan EcRs [3,6]. In fact, amino acid sequence alignment from LBD regions from representative species suggests a strong residue conservation regarding ligand binding pocket residues in close contact with steroid ligands. Furthermore, ligand binding pocket residues forming hydrogen bonds with steroid moieties—deduced from crystallographic data obtained for the tobacco budworm (*Heliothis virescens*) EcR [39,40,41]—are partially, or even fully, conserved outside ecdysozoans (Figure 1 and Table S3). Unpublished data from our group, using an annelid EcR, further suggests that full conservation of pocket residues is not strictly required to maintain Ponasterone A-induced activity (Ruivo et al, unpublished). These observations put forward a possible conservation of binding capabilities among EcRs; still, the establishment of standardized protocols and methodologies for a functional and ecotoxicological characterization of these NRs is urgently needed for the correct assessment of functional conservation across metazoan groups. This is particularly relevant for EcR, given that ecdysteroid binding to EcR requires heterodimerization with RXR, or its insect homolog USP [13]. In fact, an intricate coevolution between EcR and RXR, notably within the dimerization surfaces, has been suggested to substantiate the heterodimer as the true functional unit for ecdysteroid signaling [42]. For this reason, when using cell-based assays, the choice of cells lines must be carefully considered in parallel with the transfection strategy, to account for the availability of suitable endogenous or co-transfected RXRs/USPs [31,33].

## 3. The Ecdysone Receptor as Pesticide Target

The serendipitous discovery of a synthetic EcR ligand able to accelerate moulting paved the way for the design of dibenzoylhydrazine-based compounds, apparently exhibiting specific specificities towards lepidopteran insects (tebufenozide, methoxyfenozide and chromafenozide) or lepidopteran and coleopterans (halofenozide) [17,30]. Initially advertised as safe to non-target insects, these compounds were later shown to have significant toxicity towards mosquitoes and other Diptera [43], as well as in non-insect Entognatha hexapods [44]. In fact, EcR ligand binding affinity towards dibenzoylhydrazine compounds is highly variable among insects. For instance, lepidopteran EcR affinity towards tebufenozide is more than 100-fold higher than hemipteran EcR [30]. However, in the coleopteran *Anthonomus grandis*, the presumably coleopteran–specific halofenozide was shown to exhibit lower potency towards EcR than methoxyfenozide or tebufenozide: with estimated EC_50_ values for insecticides, derived from transactivation studies, ranging from 5 to 45 µM [38]. Still, these compounds were suggested safe and benign insecticides with respect to non-target species, including hymenopterans (bees) [17,29,37,45]. 

However, inspection of the ligand binding pocket residues highlights a full conservation of residues forming hydrogen bonds with dibenzoylhydrazine moieties across arthropod species, and a partial conservation within non-arthropod ecdysozoans and lophotrochozoans (Figure 1 and Table S3) [39]. Thus, it comes as no surprise that crustacean EcRs retain a moderate-to-high sensitivity towards dibenzoylhydrazine derivates. Using in vitro transactivation assays, tebufenozide was able to activate *Daphnia magna* EcR [32], and to moderately induce the shrimps *Americamysis bahia* [36] and *Neomysis integer* [29], and the lobster *Homarus americanus* [34]. Counterintuitively, the intermoult period of the shrimp *N. integer* was apparently unaffected by environmentally relevant concentration of tebufenozide (approx. 300 nM) [29]. This suggests that these non-steroidal agonists could act as EDCs, potentiated by the resistance towards degradation of compounds such as tebufenozide, remaining elusive with classical toxicological assessment [29,30,34,36].

## 4. Perspectives for Hazard and Risk Assessment

Environmental agencies and international organizations, such as the U.S. Environmental Protection Agency (EPA) and the Organization for Economic Co-operation and Development (OECD), already suggest a prominent role for NRs in in vitro screening. NRs-based transactivation and ligand binding assays show a high sensitivity and specificity, allowing a high-throughput testing approach that meets the 3R strategy, with a reduction of experimental animals. In the frame of the OECD, CF level 2 testing integrates receptor based assays. However, to date, standardized protocols include only mammalian ER and AR receptors. Thus, the scope of standardized NRs in vitro assays is very narrow, contrasting with data available in the scientific literature where different NRs from a wider taxonomic sample are already routinely established in different laboratories (i.e., TR, RXR, RAR, PPAR, VDR, PXR, EcR) [6]. 

Additionally, an increasing number of studies demonstrates that orthologous NRs can exhibit distinct ligand affinities across species [11]. For instance, shifts in activation profiles, when compared to non-arthropod orthologues, were reported for arthropod RXRs/USPs: with episodes of divergence or even complete loss of ligand binding capacity (i.e., constitutive activation) detected in distinct clades [46,47]. Similarly, Oestrogen Receptor (ER) orthologues from the molluscs *Octopus vulgaris* and *Aplysia californica* were found to be constitutively active and irresponsive to vertebrate hormones, whereas annelids (*Platynereis dumerilii* and *C. capitata*) respond to ER agonists similarly to vertebrates [48,49,50]. An additional, and interesting example is the Retinoic Acid Receptor (RAR) displaying high-affinity towards retinoic acid in chordates, yet low-affinity in molluscs, annelids and in the Ecdysozoa Priapulida [51,52,53,54]. 

These observations further highlight the requirements of a broader species sampling and the inadequacy of data extrapolation from a reduced number of animal models, and advocate for a shift from an “extrapolation hazard assessment paradigm” to a “precision hazard assessment paradigm”. Regarding EcR, we know today that EcR orthologs are present in more taxa than previously anticipated. Therefore, the taxonomic scope of species affected by EcR agonists/antagonists is likely to be wider. Considering the prominent utilization of NRs-based assays for hazard assessment frameworks, the central role of EcR in animal physiology, and the ability to link molecular and in vitro screening approaches with adverse outcomes on apical endpoints (i.e., moulting, growth, reproduction), we believe that the development, validation and standardization of protocols with representative EcRs is of major interest and timely.

## 5. Conclusions

Here, we put-forward the need to develop more inclusive standardized protocols, notably for non-mammalian NRs, in order to promote a more accurate phylogenetic assessment of the disruptive potential of environmental chemicals across metazoans. With this is mind, the invertebrate EcR appears as a potential candidate, due to its phylogenetic distribution and potential role in development, growth and/or reproduction. Given that OECD CF and EPA frameworks are based on tiers approaches, the development of NR-based assays should be followed by improved in silico approaches, such as quantitative structure–activity relationship (QSARS) and molecular docking, and the validation of partial and full-life cycle test with selected species. This will allow the establishment of a link between the molecular target, the biochemical changes and adversity, thus fostering the validation of new adverse outcome pathways. On the other hand, it may assist in the design of new chemicals, truly targeting the EcR of particular species of interest, mitigating potential side effects to other non-target taxa. A similar approach can be implemented for other NRs dependent-pathways.

## Supplementary Materials

The following are available online at https://www.mdpi.com/article/10.3390/toxics10010006/s1, Table S1: Ecdysone receptor sequences found within the National Center for Biotechnology Information (NCBI) database; Table S2: Ecdysone receptor characterization assays (binding and transactivation assays) found in the literature; Table S3: Substitutions of amino acid residues known to form hydrogen bonds with either Ponasterone A or synthetic ligands.
